# Identification of *Candida* Species Isolated from Renal Transplant Recipients with Candiduria

**Published:** 2016-11-01

**Authors:** M. R. Yazdani, E. Foroughifar, R. Mohammadi

**Affiliations:** 1 *Department of Infectious Diseases, Al-Zahra hospital, School of Medicine, Isfahan University of Medical Sciences, Isfahan, Iran*; 2 *Department of Medical Parasitology and Mycology, School of Medicine, Infectious Diseases and Tropical Medicine Research Center, Isfahan University of Medical Sciences, Isfahan, Iran*

**Keywords:** Identification, *Candida* species, Candiduria, Renal transplantation

## Abstract

**Background:**

Renal transplantation has long been considered the gold standard medical care for patients with end-stage renal disease. Candiduria continue to be a significant complication for renal transplant recipients. The risk of infections depends on the amount of immunosuppression and exposure to the potential pathogens.

**Objective:**

Molecular identification of *Candida* species isolated from renal transplant recipients with candiduria.

**Methods:**

Between 2009 and 2014, 62 *Candida* isolates were collected from 485 renal transplant recipients. All isolates were identified by PCR-RFLP profiles after digestion with the restriction enzyme *Msp*I.

**Results:**

*C. albicans* (44%) and* C. parapsilosis* complex (5%) had the most and the least prevalence, respectively. Male to female ratio was 26/36, ranging in age from 19 to 62 years.

**Conclusion:**

Due to the fact that candiduria is connected with increased mortality in renal transplant recipients, precise identification of *Candida* species by molecular techniques can lead to an appropriate therapy among high risk patients. *C. albicans *remains the most prevalent species isolated from renal transplant recipients, Nevertheless, the number of non-*C. albicans Candida *species looks to be emerging.

## INTRODUCTION

Renal transplantation is a well-recognized procedure for the efficient treatment of terminal renal insufficiency for thousands of patients worldwide with end-stage renal disease [1]. Kidney transplantation, being an immunosuppressed state, put the recipient at risk of a variety of viral, bacterial, and fungal infections. Urinary tract infections (UTIs) are common throughout the first several months post-transplantation [2, 3]. The risk is increased by prolonged indwelling catheterization, use of broad-spectrum antibiotics, and urinary obstruction; it is also higher in diabetic patients. The secondary obstacle may progress due to formation of a fungus ball or renal papillary necrosis [4, 5]. *Candida* species are the most common cause of fungal infections, leading to a range of life-threatening invasive to non-life-threatening mucocutaneous diseases [6]. *C. albicans* remains the main cause of candidiasis, however, the prevalence of non-*C*. *albicans* infections are increasing, consisting of 35%–65% of all *Candida* infections [7]. Considering differences in susceptibilities to antifungal drugs among *Candida *species, identification to the species level of the organism has an important role in the treatment of candidiasis [8]. This study aimed at identifying *Candida* spp. isolated from urinary tract infections in renal transplantation recipients by using molecular techniques.

## MATERIALS AND METHODS

A total of 485 renal transplant recipients (849 episodes) was registered in two university hospitals (Al-Zahra and Khorshid) in Isfahan, Central Iran, from May 2009 to August 2014. Tacrolimus, mycophenolate mofetil (CellCept), sirolimus, and cyclosporin were used for patients for immunosuppression. We had a control group including 53 kidney transplant recipients without candiduria. The samples were taken appropriately (*e.g.*, from midstream); all urine samples were examined by repeated urine culture on sabouraud glucose agar (Difco, Detroit, MI, USA), and CHROMagar Candida (Paris, France). The number of yeasts in urine specimens was counted; a count of ˃1000 colony/mL was considered “candiduria” [9]. Genomic DNA of isolates was extracted using FTA^®^ Elute MicroCards (Whatman Inc, Clifton, NJ, USA) according to the manufacturer’s guidelines [10]. 

Briefly, a loopful of a single colony was suspended in 80–100 µL of distilled water and 5 µL of the suspension was transferred to a disc of FTA card (4 mm in diameter) and incubated at 25 °C for at least 5 hrs. The dried papers were eluted in 400 µL sterile water for 10 sec; then, the paper was transferred to a new microtube containing 40 µL distilled water and incubated at 95 °C for 15 min. The paper discs were removed and the water including DNA was used for PCR and stored at 20 °C. Molecular identification of *Candida* strains was performed using an already delineated PCR-RFLP profiles [8, 11]. 

Briefly, the ITS1-5.8SrDNA-ITS2 region was amplified by a PCR mixture including of 5 µL of 10× reaction buffer, 0.4 mM dNTPs, 1.5 mM MgCl_2_, 2.5 U of Taq polymerase, 30 pmol of both ITS1 (5’-TCC GTA GGT GAA CCT GCG G-3’) and ITS4 (5’-TCC TCC GCT TAT TGA TAT GC-3’) primers [12] and 2 µL of extracted DNA in a final volume of 50 µL. The PCR cycling conditions comprised: an initial denaturation phase at 94 °C for 5 min, followed by 30 cycles of denaturation at 94 °C for 30 sec, annealing at 55 °C for 45 sec, and extension at 72 °C for 1 min, with a final extension phase at 72 °C for 7 min. During the second step, PCR products were digested with the restriction enzyme *Msp*I (Fermentas, Vilnius, Lithuania); 5 µL of each PCR amplicons and 10 µL of RFLP products were separated by gel electrophoresis on 1.5% and 2% agarose gel (containing 0.5 µg/mL ethidium bromide), respectively. 

Statistical Analysis

Data were analyzed with SPSS^®^ for Windows^®^ ver 14.0. Comparison of species distribution and end-stage renal disease was adjusted using Fisher’s exact test and *Mann-Whitney U *test. A p value <0.05 was considered significant.

## RESULTS

**Figure 1:**
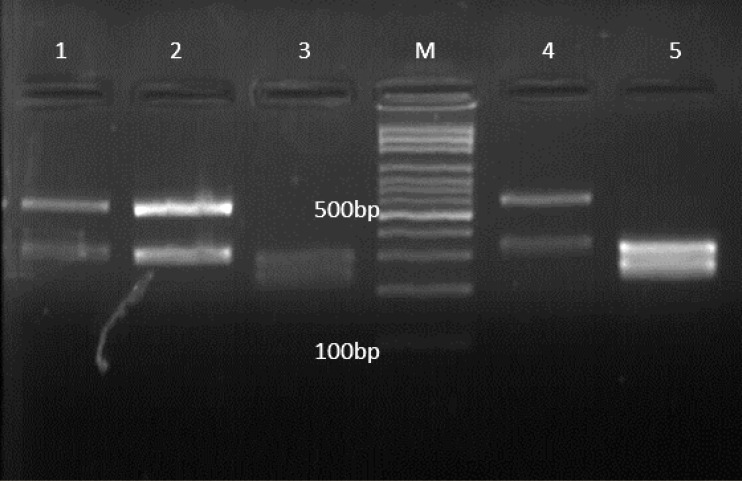
Agarose gel electrophoresis of ITS-PCR products of various *Candida* spp. after digestion with *Msp*I. Lanes 1, 2, and 4 are *C. glabrata*, Lanes 3, and 5 are *C. albicans*, and Lane M is a 100-bp DNA size marker

Sixty-two patients were diagnosed with candiduria (colony counts >1000/mL) and all isolates were identified by PCR-RFLP profile on agarose gel (Fig 1). *C. albicans* (44%) and* C. parapsilosis* complex (5%) had the most and the least prevalence, respectively (Table 1). Twenty-six patients were male (42%) and 36 (58%) were female, ranging in age from 19 to 62 years (Table 2). Diabetes mellitus (DM) and high blood pressure (HBP) were the two leading causes of end-stage renal disease among patients with candiduria (Table 3). *C. albicans* was the most prevalent species isolated from diabetic patients (65%), followed by *C. tropicalis* (15%), and *C. glabrata* (15%). Twenty-eight (45%) patients were hospitalized in ICU, 18 (29%) in transplantation ward, and 16 (26%) in general medicine ward. Fourteen (22.5%) patients had lower urinary tract symptoms (LUTS) such as dysuria, frequency, and incomplete voiding; 6 (10%) patients had upper urinary tract symptoms (UUTS) including fever, chills, pain and tenderness, nausea, and vomiting, while 42 (68%) were asymptomatic. Table 4 summarizes the association between patients with candiduria and body mass index (BMI) in the present study. The serum creatinine level was 0.7 to 1.3 mg/dL for men and 0.6 to 1.1 mg/dL for women except for eight (13%) patients (Table5). In two (3%) patients, we had transplant rejection. In the control group, we had six (11%) cases of elevated serum creatinine level, two (4%) of transplant rejection, and three (6%) of death. Pneumonia (in two patients) and gastrointestinal bleeding (GIB) (in one patient) were the causes of death in this group. All patients who died (a male and two females) were hospitalized in ICU. 

**Table 1:** Distribution of *Candida* spp. among renal transplant recipients with candiduria

*Candida* spp.	Frequency, n (%)		
	Male	Female	Total
*C. albicans*	10 (39)	17 (47)	27 (44)
*C. glabrata*	8 (31)	8 (22)	16 (26)
*C. tropicalis*	3 (12)	4 (11)	7 (11)
*C. krusei*	3 (12)	2 (6)	5 (8)
*C. parapsilosis* complex	1 (4)	2 (6)	3 (5)
Mixed infection	1 (4)	3 (8)	4 (7)
Total	26 (100)	36 (100)	62 (100)

**Table 2:** Age distribution of patients stratified by sex

Age Group	Male	Female	Total number (%)
10–19	1	0	1 (2)
20–29	5	3	8 (13)
30–39	2	9	11 (18)
40–49	8	9	17 (27)
50–59	8	12	20 (32)
60–70	2	3	5 (8)
Total	26	36	62 (100)

**Table 3:** Causes of end-stage renal disease (ESRD) among patients with candiduria

Cause of ESRD	Frequency (%)	*Candida *spp.
Diabetes mellitus	26 (42)	*C. albicans* (n=17), *C. tropicalis* (n=4), *C. glabrata* (n=4), *C. krusei *(n=1)
Hypertension	15 (24)	*C. albicans* (n=6), *C. glabrata* (n=5), *C. krusei* (n=3), *C. tropicalis* (n=1)
Cyst	6 (10)	*C. glabrata *(n=4), *C. krusei *(n=1), *C. albicans* (n=1)
Kidney stone	6 (10)	*C. albicans *(n=2), *C. tropicalis *(n=1), Mixed (n=3)
Glomerulonephritis	4 (7)	*C. albicans* (n=1), *C. tropicalis* (n=1), *C. glabrata* (n=1), *C. parapsilosis* complex (n=1)
Unknown	5 (8)	*C. glabrata* (n=2), *C. parapsilosis* complex (n=2), Mixed (n=1)
Total	62 (100)	*C. albicans* (n=27), *C. glabrata* (n=16),* C. tropicalis* (n=7),* C. krusei *(n=5),* C. parapsilosis* complex (n=3), Mixed (n=4)

**Table 4:** Distribution of body mass index in patients with candiduria

Sex	Body Mass Index (kg/m^2^), n (%)			Total
	˂25	25–30	˃30	
Male	11 (18)	11 (18)	4 (6)	26 (42)
Female	19 (31)	14 (23)	3 (5)	36 (58)
Total	30 (48)	25 (40)	7 (11)	62 (100)

**Table 5:** The association between elevated serum creatinine level and *Candida *spp. among patients

Patient #	Cr level (from)	Cr level (to)	*Candida *spp.	Outcome
1	1.1	4	*C. albicans*	Lowered back to normal level
2	0.9	3.5	*C. albicans*	Lowered back to normal level
3	1.3	3	*C. albicans*	Lowered back to normal level
4	1	6.5	*C. albicans*	Transplant rejected
5	1.2	5.8	*C. albicans*	Transplant rejected
6	0.9	3.1	*C. glabrata*	Lowered back to normal level
7	1.3	3.8	*C. glabrata*	Lowered back to normal level
8	1.3	4.1	*C. tropicalis*	Lowered back to normal level

## DISCUSSION

Infections involve 50%–75% of renal transplant recipients with mortality rate ranging from 20% to 60% [13]. *Candida* species are the most common cause of urinary tract fungal infections [14]. The interpretation of candiduria is ambiguous depending on the patient’s conditions, changeable cut-off definitions and unpredictable culture results. Safdar, *et al* [15], reported that 11% of renal transplant recipients had at least one episode of candiduria within two months of transplantation. They showed that in renal transplant recipients, the first episode of candiduria appeared after a median of 54 days of renal transplantation (range: 0–97 months). In the present study, 22 (36%) patients had one episode of candiduria during the first month, 26 (42%) had after two months, and 14 (23%) after six months. Candiduria is very frequent in hospitalized patients and is predominantly asymptomatic. This means a large number of infected patients would have no dysuria, fever, or other urinary tract-related complaints like leukocyturia. There is evidence showing that the frequency of candiduria is associated with antibiotic usage [16]. The incidence of candiduria also varies in different parts of hospitals, being most prevalent in ICUs [17], in accordance with the present study. Pyelonephritis, epididymitis, and prostatitis can also lead to candiduria, especially in old immunosuppressed men [18]. We recognized pyelonephritis in five (8%) patients with upper urinary tract symptoms, but there were no signs of epididymitis or prostatitis in our patients. In comparison with bacteriuria, the majority of patients with candiduria do not present with accompanying septicemia. Some studies showed that 1%–8% of patients with candiduria also present with candidemia. We found no one with candidemia. The risk factors for candiduria include abdominal surgery, urinary tract anatomical abnormalities, comorbidities, admission to ICU, diabetes mellitus, urinary catheterization, use of broad-spectrum antibiotics, female sex, and increased age [19-21]. For example, Richards, *et al* [22], showed that urinary fungal infections take place more often in patients with urinary catheters than in those without urinary catheters (40% *vs* 22%). In contrast to our findings, Harris, *et al* [23], showed that fluconazole usage is a risk factor for *C. glabrata-*mediated diseases. The progression of invasive candidiasis depends on the virulence of the isolate and disability of host defenses [24]. In accordance with the present study, many investigations show that *C. albicans* accounts for 50%–70% of candidurias; it is followed by *C. glabrata, C. tropicalis, *and* C. krusei* [25-27]. As opposed to the present study, in many investigations, *C. parapsilosis* complex has become a principal *Candida* species that is connected to candidiasis, including candiduria [28, 29]. In conformity with our own investigation, some studies showed that 5%–8% of patients with candiduria have two or more *Candida* species concurrently [6, 21]. In the present study, the male to female ratio was 42/58. The presence of *Candida *in vagina may explain the greater incidence of candiduria in women. The presence of yeast in the urine (positive urine culture), must be evaluated for making an appropriate decision about the necessity of antifungal therapy. *Candida* species affect different groups, so different treatment regimens may be needed. High-risk patients must be treated with antifungal agents rapidly. Although fluconazole is the main antifungal agent used in patients with candiduria, resistant isolates to fluconazole are emerging. Although *C. albicans *remains the principal species isolated from candiduria, non-*C*. *albicans *species are increasing. Due to the fact that candiduria is associated with increased mortality, precise identification of *Candida* species by molecular techniques can lead to a more appropriate treatment among high risk patients, as we did in the present study.
